# Psychiatric-Predominant Wernicke Encephalopathy With Normal Blood Thiamine Levels: A Case Report

**DOI:** 10.7759/cureus.105343

**Published:** 2026-03-16

**Authors:** Shota Inada, Yuhei Mori, Shin Kumashiro, Chikako Kaneko, Ryo Kanamori, Itaru Miura

**Affiliations:** 1 Department of Neuropsychiatry, Fukushima Medical University, Fukushima, JPN; 2 Department of Psychiatry, Haryugaoka Hospital, Koriyama, JPN; 3 Department of Neurology, Southern Tohoku General Hospital, Koriyama, JPN

**Keywords:** alcohol use disorder, high-dose thiamine, normal thiamine levels, psychiatric symptoms, wernicke encephalopathy

## Abstract

Wernicke encephalopathy (WE), caused by thiamine deficiency in patients with chronic alcohol use, may progress to Korsakoff syndrome if untreated. Diagnosis is challenging and often missed because the classic triad can be incomplete and blood thiamine levels can be normal. A 56-year-old woman with 40-60 g of pure alcohol consumption daily since her 20s and a previous psychiatric hospitalization for alcohol dependence at the age of 50 years developed hallucinations, delusions, agitation, and cognitive decline. One month later, she fractured her left femoral neck at home, for which she underwent hemiarthroplasty at a general hospital. Although no clear alcohol withdrawal symptoms were observed, the patient was unable to engage in meaningful conversations and was diagnosed with alcohol-related dementia. Antipsychotic treatment was ineffective, and she was transferred to our hospital. Upon admission, her Hasegawa Dementia Scale-Revised (HDS-R) score was 1. Neurological examination, brain computed tomography (CT), and laboratory tests, including blood thiamine levels, appeared normal. Based on the patient’s clinical course, WE was suspected. High-dose intravenous thiamine (400 mg/day) was administered for five days, followed by oral thiamine. Her psychiatric symptoms and cognitive function rapidly improved, and the HDS-R score increased to 27 by day 17. The patient was diagnosed with WE. WE diagnosis can be difficult, particularly in patients with alcohol use disorder and atypical presentations. High-dose intravenous thiamine is safe and may serve as an intervention with diagnostic value when WE cannot be excluded.

## Introduction

Wernicke encephalopathy (WE), a neurological disorder caused by thiamine (vitamin B1, VB1) deficiency, occurs predominantly in individuals with chronic heavy alcohol consumption [1]. Thiamine is an essential cofactor in cerebral energy metabolism, and its deficiency can impair neuronal function, leading to encephalopathy and neuropsychiatric symptoms. If left untreated, WE has a high risk of progression to Korsakoff syndrome with associated mortality. Therefore, early administration of adequate intravenous thiamine is essential. However, the classic triad of WE of altered mental status, ophthalmoplegia, and gait ataxia is rarely fully present, and the disorder may develop even when blood thiamine levels are within normal limits, making diagnosis challenging [2]. Furthermore, WE can cause disturbances in consciousness and varying psychiatric symptoms, including memory impairment, affective changes, and psychosis [1]. These atypical and heterogeneous clinical features further complicate diagnosis. Psychiatrists often encounter patients with heavy alcohol use and psychiatric symptoms; however, WE is frequently overlooked.

Here, we describe the case of a patient with an alcohol-related disorder who presented with predominantly psychiatric symptoms and normal blood thiamine levels and was treated with thiamine approximately two months after the onset of symptoms. Administration of high-dose IV thiamine resulted in marked clinical improvement. This case demonstrated the importance of considering WE in the differential diagnosis and the utility of intravenous thiamine therapy. This study followed the ethical guidelines of the Fukushima Medical University Hospital and conformed to the Declaration of Helsinki.

## Case presentation

**Figure 1:**
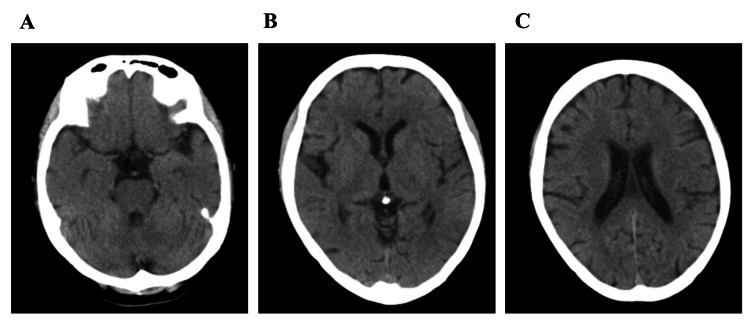
Head computed tomography findings on admission Axial head computed tomography (CT) images obtained on admission at the level of the basal cisterns (A), basal ganglia (B), and lateral ventricles (C). No apparent cerebral atrophy, ventricular enlargement, intracranial hemorrhage, mass lesion, or significant white matter abnormalities were observed. These findings indicated no obvious structural brain lesion on the initial CT scan

**Figure 2:**
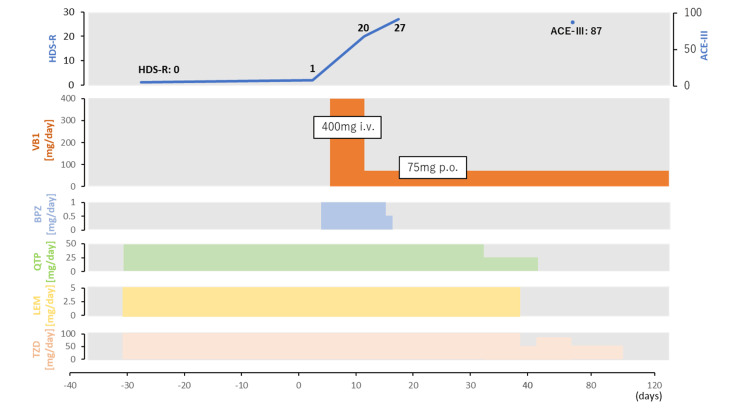
Time course of medical interventions and changes in cognitive function ACE-III: Addenbrooke’s Cognitive Examination-III; HDS-R: Hasegawa Dementia Scale-Revised; BPZ: brexpiprazole; QTP: quetiapine; LEM: lemborexant; TZD: trazodone The figure shows the clinical course from the prodromal period to treatment response, including changes in cognitive test scores and psychotropic medication use. Responses to psychotropic medications were limited. Intravenous vitamin B1 (VB1) was initiated approximately two months after symptom onset and was followed by marked improvement in cognitive function, with HDS-R improving from 0 to 27 and ACE-III reaching 87 on hospital day 67. Cognitive improvement was maintained after tapering or discontinuation of psychotropic medications

A 56-year-old woman was admitted to our psychiatric hospital with acute psychotic symptoms and cognitive decline. At 17 years of age, she was diagnosed with anorexia nervosa and achieved remission after inpatient treatment. From her 20s onward, she had consumed approximately three to four units of alcohol daily. At 50 years of age, she was hospitalized for alcohol dependence but discontinued treatment independently after discharge. Until approximately two months prior to admission, she had functioned independently with her husband. When her husband was hospitalized for lung cancer, she remained home alone. She had been left alone at home for approximately two months before admission; however, the details of her alcohol consumption during this period could not be determined. After discharge, he noticed that his wife was unable to sustain coherent conversation, expressed delusional ideas, and was unable to perform household chores. Although her husband encouraged her to seek psychiatric care, she refused treatment. Approximately 10 days later, she sustained a left femoral neck fracture at home and underwent inpatient femoral head replacement surgery at a general hospital. Alcohol withdrawal symptoms were not observed; however, confused speech and behavior were observed. She scored 0 points on the Hasegawa Dementia Scale-Revised (HDS-R) [3]. Because a brain magnetic resonance imaging (MRI) was not available at our facility, the neuroimaging evaluation was limited to head computed tomography (CT). Head CT showed no apparent cerebral atrophy, ventricular enlargement, or white matter lesions (Figure 1), and blood thiamine (VB1) levels were within the normal range. On the basis of these findings, the patient was diagnosed with alcohol-related dementia. Oral quetiapine (50 mg/day), trazodone (100 mg/day), and lemborexant (5 mg/day) were initiated; however, their therapeutic effects were limited. The patient was transferred to our psychiatric hospital for further evaluation and environmental adjustments (Figure 2).

On admission, her response was markedly slow, and no valid answers to questioning were obtained, except for her date of birth and the current date. Ocular motor, finger-to-nose, and rapid alternating movement tests were normal. Her height was 153 cm, body weight was 32 kg, and body mass index (BMI) was approximately 13.8 kg/m², indicating severe emaciation. Laboratory findings revealed albumin (4.2 g/dL), cholinesterase (218 U/L), blood glucose (78 mg/dL), and thiamine (33 ng/mL), all within the normal range. Mild elevations in the liver enzyme levels were noted: aspartate aminotransferase (AST), 32 U/L; and alanine aminotransferase (ALT), 47 U/L. Complete blood count, renal function, thyroid function, electrocardiography, and chest radiography results were unremarkable (Table 1). 

**Table 1:** Blood test results CRP: C-reactive protein Laboratory findings showed no marked inflammatory response or severe hepatic, renal, or nutritional abnormalities at presentation. Mild elevations in platelet count, aspartate aminotransferase, and alanine aminotransferase were noted

Test item	On admission	Reference range
White blood cell count	4790 cells/μL	3300-8600 cells/μL
Hemoglobin	12.4 g/dL	11.6-14.8 g/dL
Platelet count	44.4 × 10^4^/μL	15.8-34.8 × 10^4^/μL
Albumin	4.2 g/dL	4.1-5.1 g/dL
Cholinesterase	218 U/L	201-421 U/L
Aspartate aminotransferase	32 U/L	13-30 U/L
Alanine aminotransferase	47 U/L	7-23 U/L
Alkaline phosphatase	65 U/L	106-322 U/L
γ-glutamyltransferase	18 U/L	9-32 U/L
Total bilirubin	0.37 mg/dL	0.4-1.5 mg/dL
Creatine kinase	20 U/L	41-153 U/L
Urea nitrogen	17.7 mg/dL	8-20 mg/dL
Creatinine	0.66 mg/dL	0.46-0.79 mg/dL
Glucose	78 mg/dL	73-109 mg/dL
CRP	0.1 mg/dL	<0.3 mg/dL
Sodium	144 mmol/L	138-145 mmol/L
Potassium	4.4 mmol/L	3.6-4.8 mmol/L
Chloride	97 mmol/L	101-108 mmol/L
Vitamin B1	33 ng/mL	21.3-81.9 ng/mL
Thyroid-stimulating hormone	3.09 μIU/mL	0.35-4.94 μIU/mL
Free triiodothyronine	2.38 pg/mL	1.71-3.71 pg/mL
Free thyroxine	0.88 ng/dL	0.70-1.48 ng/dL

**Figure 3:**
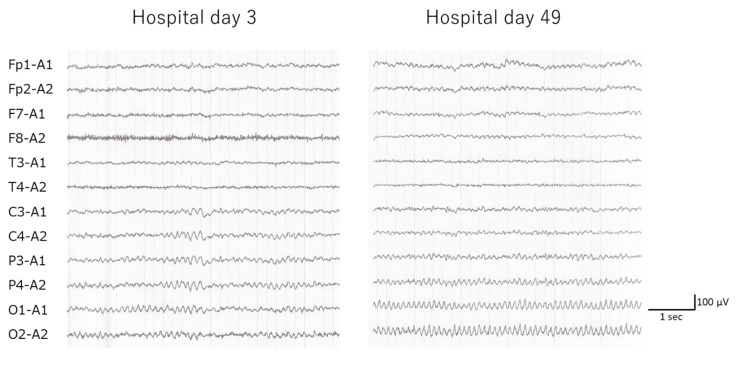
Comparison of EEG findings before and after VB1 administration EEG: electroencephalography; VB1: thiamine Representative EEG recordings obtained on hospital day 3 (left) and hospital day 49 (right) are shown. After VB1 administration, background activity became faster and more organized, with clearer posterior dominant occipital rhythm, indicating electrophysiological improvement in parallel with the patient’s clinical recovery. No epileptiform discharges were observed in either recording

Electroencephalography (EEG) demonstrated a background rhythm of predominantly slow alpha activity around 8 Hz, distributed from the central to occipital regions, with mildly poor organization. No definite epileptiform discharges were observed (Figure 3). 

The HDS-R score was 1. After admission, she continued to exhibit confused speech and behavior. Oral brexpiprazole (1 mg/day) was initiated on hospital day five. Given the presence of features atypical of alcohol-related dementia, including diurnal fluctuations and rapid progression in psychiatric symptoms over several months, and the long history of heavy alcohol consumption, WE was suspected. Intravenous thiamine was administered (200 mg twice daily for five days) starting on hospital day seven. By hospital day 9, her communication had markedly improved, and on hospital day 10, her HDS-R score had increased to 20 points. However, some confused statements persisted. On day 11, a neurologist observed no abnormalities in muscle strength, tendon reflexes, position sense, or finger-to-nose testing. Based on the clinical course and favorable response to thiamine treatment, the diagnosis of WE was made, and the thiamine was switched to oral administration (75 mg/day). By hospital day 16, the HDS-R score had further improved to 27 points, and most of the confused speech and behavior had resolved. On hospital day 19, laboratory testing revealed elevated liver enzyme levels (AST, 118 U/L; ALT, 245 U/L), raising the suspicion of drug-induced liver injury caused by brexpiprazole. The medication was discontinued, and the liver enzyme levels normalized. On hospital day 49, follow-up EEG demonstrated a well-organized basal rhythm dominated by 9-10 Hz alpha activity in the occipital regions (Figure 2). The patient and her husband were informed of the diagnosis and advised to modify their drinking behaviors. The patient demonstrated limited insight, and her husband appeared somewhat resigned. Subsequently, the patient appeared to exhibit impaired insight and poor control over alcohol cravings, including lying to obtain permission for overnight stays, secretly consuming alcohol during these outings, and denying alcohol use. Lemborexant, quetiapine, and trazodone were gradually discontinued on hospital days 39, 46, and 102, respectively, without recurrence of psychiatric symptoms. The Addenbrooke’s Cognitive Examination-III (ACE III) score [4] was 87 points on hospital day 67. The patient was discharged on day 108 of hospitalization. Her body weight had increased to 37.4 kg (BMI, approximately 16.0 kg/m²). Two months after discharge, the patient continued outpatient follow-up with no recurrence of psychiatric symptoms. Given that the amount of alcohol consumption cannot be determined, prophylactic oral thiamine at 75 mg/day has been continued along with motivational interventions for alcohol use disorder, including the use of drinking records.

## Discussion

WE is classically characterized by a triad of altered mental status, ophthalmoplegia, and gait ataxia; however, the reported frequencies of these symptoms are 82%, 29%, and 23%, respectively, and only 10-16% of patients present with the complete triad [1,2]. Therefore, the absence of the full triad does not exclude this diagnosis. The Caine criteria suggest that WE can be diagnosed in patients with chronic alcohol use when at least two of the following features are present: malnutrition, ocular motor dysfunction, cerebellar signs, and altered mental status, or mild memory impairment, with a reported sensitivity of 85-94% [5,6]. Regardless, clinical diagnosis requires a comprehensive assessment rather than reliance on a single criterion. The reported clinical prevalence of WE (0.04-0.13%) is markedly lower than that identified in autopsy studies of the general population (0.8-2.8%), suggesting substantial underdiagnosis, particularly among patients with alcohol use disorder. Another previous study reported that 12% of inpatients receiving psychiatric care met the Caine criteria [7], indicating that WE is even more frequently overlooked in psychiatric practice.

The present patient had a history of chronic heavy alcohol consumption and exhibited an altered mental status at presentation, without cerebellar signs or ocular motor dysfunction. Marked underweight upon admission and subsequent weight gain during hospitalization suggested prolonged malnutrition, thereby fulfilling the Caine criteria retrospectively. The psychiatric and cognitive symptoms that had persisted for approximately two months improved markedly after thiamine administration and did not recur after the discontinuation of psychotropic medications. These findings strongly supported the diagnosis of WE.

Blood thiamine levels and brain MRI are commonly used as diagnostic tools; however, these are not sufficient to exclude WE. In patients with chronic alcohol use, increased thiamine consumption and reduced expression of thiamine transporters (THTR1 and THTR2) in the intestine and blood-brain barrier impair thiamine delivery to the brain [8]. Downregulation of THTR2 has been particularly implicated in the development of WE, even when blood thiamine levels are within the normal range [9], and several such cases have been reported [10,11]. In addition, measured blood thiamine levels may not accurately reflect tissue-level or cerebral thiamine availability, because circulating concentrations do not necessarily indicate intracellular utilization. Therefore, normal blood thiamine levels should be interpreted cautiously in patients with suspected WE. Although typical MRI findings include symmetrical hyperintensities in the periventricular regions, with a reported sensitivity of 53% and specificity of 93% [12], abnormalities are less frequently observed in patients with alcohol use disorder [13], and imaging findings may be unremarkable [14]. EEG abnormalities, most commonly diffuse slowing, have occurred in approximately half of patients with WE [14]. Although nonspecific, the improvement in EEG findings after thiamine administration in the present case provided evidence for cerebral functional recovery.

WE is associated with a poor prognosis if left untreated, with irreversible neurological damage and mortality reported in approximately 20% of cases [14]. High-dose IV thiamine therapy improves neurological and cognitive symptoms in approximately 90% of patients [15]. Although no consensus exists on the optimal dose, intravenous administration of thiamine two to three times daily is recommended because of its short half-life and impaired intestinal absorption in patients with chronic alcohol use [14]. Daily doses of 100-500 mg are commonly used [16], and higher doses have been recommended [1,17]. We administered 400 mg/day divided into two doses. Adverse events associated with intravenous thiamine, including anaphylaxis, are extremely rare (with only four cases reported per one million administrations in the United Kingdom) [17,18]. When weighed against the risk of untreated WE, high-dose IV thiamine is a safe intervention.

Our study had limitations. First, a brain MRI was not performed, which may have limited the exclusion of other neurological conditions. Second, this was a single-case report, and the findings may not be generalizable. Third, blood thiamine levels did not necessarily reflect cerebral thiamine availability, and the timing and method of measurement may have influenced the results. In addition, although the diagnosis was supported by a marked response to thiamine, the therapeutic response alone did not definitively confirm the diagnosis. Finally, the EEG findings were nonspecific.

## Conclusions

Atypical WE is difficult to diagnose when presenting with predominantly psychiatric symptoms or normal blood thiamine levels and is easily overlooked in psychiatric settings. If left untreated, WE may result in irreversible neurological damage or death, whereas high-dose intravenous thiamine administration may lead to substantial clinical improvement. This case demonstrates that WE can remain reversible, even in patients with predominantly psychiatric symptoms, normal blood thiamine levels, and a prolonged clinical course. In patients with heavy alcohol use who present with psychiatric symptoms, WE should be included in the differential diagnosis and high-dose intravenous thiamine should be considered.
